# Assessment of the Mechanical Support Characteristics of a Light and Wearable Robotic Exoskeleton Prototype Applied to Upper Limb Rehabilitation

**DOI:** 10.3390/s22113999

**Published:** 2022-05-25

**Authors:** Manuel Andrés Vélez-Guerrero, Mauro Callejas-Cuervo, Juan C. Álvarez, Stefano Mazzoleni

**Affiliations:** 1 Software Research Group, Universidad Pedagógica y Tecnológica de Colombia, Tunja 150002, Colombia; mauro.callejas@uptc.edu.co; 2 Multisensor Systems and Robotics Group (SiMuR), Department of Electrical, Electronic, Computer and Systems Engineering, University of Oviedo, C/Pedro Puig Adam, 33203 Gijón, Spain; juan@uniovi.es; 3 Department of Electrical and Information Engineering, Polytechnic University of Bari, 70126 Bari, Italy; stefano.mazzoleni@poliba.it

**Keywords:** robotic exoskeletons, soft materials, wearable devices, upper limbs, rehabilitation, device testing, mechanical support, optical tracking, optical motion capture

## Abstract

Robotic exoskeletons are active devices that assist or counteract the movements of the body limbs in a variety of tasks, including in industrial environments or rehabilitation processes. With the introduction of textile and soft materials in these devices, the effective motion transmission, mechanical support of the limbs, and resistance to physical disturbances are some of the most desirable structural features. This paper proposes an evaluation protocol and assesses the mechanical support properties of a servo-controlled robotic exoskeleton prototype for rehabilitation in upper limbs. Since this prototype was built from soft materials, it is necessary to evaluate the mechanical behavior in the areas that support the arm. Some of the rehabilitation-supporting movements such as elbow flexion and extension, as well as increased muscle tone (spasticity), are emulated. Measurements are taken using the reference supplied to the system’s control stage and then compared with an external high-precision optical tracking system. As a result, it is evidenced that the use of soft materials provides satisfactory outcomes in the motion transfer and support to the limb. In addition, this study lays the groundwork for a future assessment of the prototype in a controlled laboratory environment using human test subjects.

## 1. Introduction

Robotic exoskeletons, also called exosuits, are complex devices that can assist, amplify, substitute, or counteract the movements of human body parts [1]. They are usually located on the different joints of the upper or lower extremities, interacting with the human musculoskeletal structure [2]. Robotic exoskeletons can have a variety of mechanical structures, actuators, sensors, and electronic control systems to perform a specific motor function, disposed of according to the design criteria defined in specific cases [3]. The most common applications of robotic exoskeletons include motion support in industrial or work environments, haptic interaction with virtual systems, joint assessment, or even rehabilitation process support systems [4,5]. Hence, robotic exoskeletons have become an active research area cross-cutting engineering, industry, and health sciences.

Within robotic exoskeletons, there are several classifications that group devices according to the actuation mode, employed materials, physical characteristics, control techniques, and their final utility [6]. 

In summary, robotic exoskeletons can have a single or multiple degrees of freedom [7,8], consisting of different construction materials such as metal alloys, textiles, or foams [9], and have a passive or active assistance system with external actuators [10]. Research into new actuation techniques and manufacturing materials has produced a new generation of soft exoskeletons that are particularly applicable in rehabilitation [11,12], including cable-actuated prototypes [13] with soft or flexible support structures [14]. The use of soft parts in combination with the rigid segments of conventional exoskeletons has enabled an improved ergonomic design adapted to individual needs, contributing to the development of more compact, lighter, and more wearable systems [6,15,16].

In robotic exoskeletons used for rehabilitation, regardless of the materials used for their construction, special attention must be given to their physical properties, as they interact directly with the human body and influence its health condition [17]. Since exoskeletons are intended as clinical devices, and considering the abnormalities or deficiencies of each patient’s musculoskeletal and nervous systems, pathologies such as variations in muscle tone and the management of spastic conditions raise the safety and reliability expectations that should be adopted concerning the physical design of robotic exoskeletons [18,19,20].

Considering the above, some of the most relevant aspects are an effective range of motion, performance metrics on the executed movement, low inertia, safety of use, and adaptability of the exoskeletons [4,21,22]. Some important aspects to consider when using soft building materials include effective motion transfer, mechanical limb support, stability during movement, deformation, and resistance to disturbances [23,24]. This study aims to evaluate the mechanical support properties of a prototype upper limb robotic exoskeleton when incorporating soft or flexible materials into critical parts of the design. This research is in response to the growing need to improve the physical capabilities of robotic exoskeletons to assist in rehabilitation processes.

This study evaluates the mechanical performance of the prototype soft straps in a laboratory environment by simulating flexion and extension movements of the elbow joint and an increased muscle tone (spasticity). For this purpose, information from the exoskeleton’s internal sensor system and an external optical tracking system (OTS) is used to trace the position and orientation of the system with high reliability and accuracy in the controlled test environment [25].

This article is divided into several consecutive sections. Section 2 presents the background that addresses the physical evaluation of robotic exoskeletons. Section 3 describes the materials used to develop the research, focusing on the required technological scenario. Section 4 presents the methodology and design of the experimental protocol, including a description of the method used to collect and analyze the data. Section 5 summarizes and analyzes the results, followed by a discussion of the results. Finally, Section 6 presents the conclusions and opportunities for future work arising from this research.

## 2. Related Works

To provide background on the key elements addressed in this research, the following is a brief compilation of relevant studies discussing the mechanical properties of robotic exoskeletons and the evaluation procedures used to assess the physical support properties of the devices presented in each study.

### 2.1. Mechanical Properties of the Robotic Exoskeletons

As mentioned above, robotic exoskeletons can be passive or active, including one or several degrees of freedom [26]. In general, active exoskeletons typically have a mechanical actuator and are manufactured with different materials; some of them are rigid, but others include softer, flexible, and more ergonomic alternatives [9].

Some of the studies offer different approaches to manufacturing techniques and actuation methods. In the overview presented in [15], robotic exoskeletons designed with a mix of soft materials are highlighted as an alternative to standard rigid exoskeletons, as their weight or size might be inadequate in some cases [27]. Constantly growing alternatives to conventional exoskeleton developments are using soft or semi-rigid elements [15,28,29]. Advances in materials have driven new forms of mechanical actuation, making other techniques such as pneumatic [30] or cable-driven systems [31] more popular, as conventional servo-based systems can experience coupling issues with soft materials.

As a particular case study, the research conducted by Seth et al. [32] highlights a soft robotic exoskeleton design for upper limb rehabilitation, especially for elbow motion assistance. It stands out due to the versatility of the proposed materials to be used as an alternative to fully rigid systems. Another particular case study is presented in [33], where an upper limb exoskeleton is implemented using an unfoldable textile that transfers the motion to the limb. In this case, the exoskeleton structure itself becomes a pneumatic actuator, allowing simultaneous support and movement of the limb.

However, other studies such as [27,34] emphasize that some of the mechanical properties needed to assist human body limbs have to be satisfactory regarding the effective motion transfer. Therefore, developing effective systems is challenging when soft or flexible solutions are used.

### 2.2. Mechanical Evaluation of Robotic Exoskeletons

The evaluation of assistive robotic exoskeletons, whether for rehabilitation or industrial use, is critical for effective user interaction and the continuous improvement of the technologies involved, including the various techniques, methods, and processes employed in their development [35]. Recently, there has been documented use of methodologies for motion evaluation of this and other types of devices [36], where the use of optical systems excels in providing motion information in space with high accuracy and versatility [37].

The use of optical measurement systems in human motion analysis offers advantages such as rapid data acquisition and high resolution in the digital reconstruction of motion information [38]. Although there are some limitations, such as occlusion, loss of markers, or difficulties in reconstructing trajectories in certain environments [25], the use of optical systems is a gold standard for conducting motion research in controlled environments such as laboratories and specialized facilities [39].

Some studies are prominent in handling the mechanical evaluation procedures and the performance of robotic exoskeletons [40]. For example, in [41], a protocol is presented to determine the mechanical support of a robotic exoskeleton called Proto-MATE. This system is evaluated using different parameters involving users in workplace conditions while executing predefined motion routines. Related research focuses on robotic exoskeletons assessment with industrial applications [42], proposing a method to evaluate a robotic system in a tridimensional space using optical tracking cameras. It is worth pointing out that this study evaluates the motion characteristics of a hybrid exoskeleton with rigid and soft materials. The performed movements are focused on load-lifting tasks, evidencing that the hydraulic actuation system is efficient and stable during the test routines.

Some papers address the evaluation of mechanical support of the upper limbs when using a soft exoskeleton, specifically when employing textiles [43,44]. In such cases, an optical motion capture system is used. As a result, it is shown that the exoskeleton can provide comprehensive upper limb support despite some drawbacks in the effective motion transfer from the exoskeleton to the limbs when using soft parts. It is highlighted that the use of soft materials enables better ergonomics of the manufactured device, which leads to the success of the proposed development.

Finally, studies such as those shown in [45,46], have focused on the comprehensive evaluation of assistive devices while the user is wearing them. Although not a direct assessment of mechanical components, motion capture systems can also aid in the modeling and subsequent design and improvement of these systems. Considering the above, it is concluded that the use of optical motion capture technologies under monitored evaluation and measurement environments allows for determining the mechanical and motion properties of various systems, specifically robotic exoskeletons made of flexible or soft materials and their interaction with the limbs of the human body. 

## 3. Materials

This section presents the materials used for the development of this research. Mainly, the materials used consist of an upper limb robotic exoskeleton prototype, a dummy arm that simulates the limb to be actuated by the exoskeleton (which is anatomically equivalent to the human model), and an optical motion capture system arranged on an experimental stage. This set of elements will allow us to analyze the motion of the robotic exoskeleton prototype, as well as the mechanical changes produced on the flexible or textile materials and their relationship with the actuated limb. Each element mentioned above is described in detail below.

### 3.1. Robotic Exoskeleton Prototype

**Figure 1:**
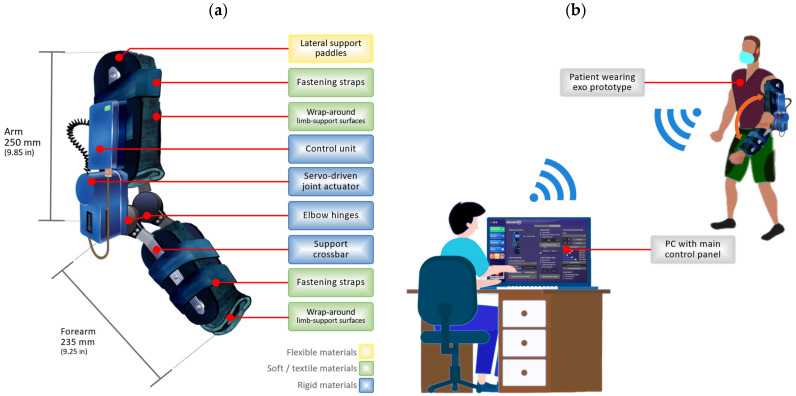
(**a**) Robotic exoskeleton prototype, dimensions, and types of materials used for the main components manufacturing, (**b**) conceptual scheme of operation and control of the system.

The robotic exoskeleton prototype, as shown in Figure 1a, is a soft support structure along with a 1-degree-of-freedom (1-DoF) servo-driven rigid joint designed to actively or resistively assist flexion and extension motion about the elbow joint. This prototype and its servo-driven joint have been previously described in [47,48]. Functionally, motion control over the prototype is performed remotely and wirelessly using a networked computer, as illustrated in Figure 1b. The different control modes allow a user interface to interact directly with the behavior of the robotic exoskeleton, using preset or remote-controlled movements in real-time.

Physically, the exoskeleton is constructed with a combination of rigid, flexible, and soft (textile) materials, favored by modular construction and lower cost compared to other developments. The soft wraps and textile surfaces hold and support the human arm, while the exoskeleton assists or opposes movement in the rehabilitation process. The wrap-around surfaces on the limbs are made of polychloroprene, but other materials such as rubber or nylon are also used for structural support elements such as the fastening straps.

Due to their physical properties, the wrap-around structures supporting the limb weight can lead to mechanical deformations caused by gravity and external mechanical forces. Likewise, the additional mechanisms for attaching the exoskeleton to the limb (lateral support paddles and fastening straps) are susceptible to strain, as they are attached to the limb in the middle of the execution of the movements as a result of the direct action of the servo actuator. Although the forces experienced by the robotic prototype depend on the spatial positioning of the arm and its load characteristics, the robotic exoskeleton is set to allow the load exerts a maximum physical stress, taking the device test to its limits in any scenario. 

The motion generated by the actuator is transmitted laterally by flexible support paddles made of thermoformed plastic (ABS) fixed to a rigid hinge, the center of which is connected to the main drive shaft of the servo articulation.

The dimensions of the prototype are compact, with an arm segment length of 250 mm, and a forearm segment length of 235 mm, corresponding anatomically to the average adult dimensions. The joint amplitude ranges from 0° (maximum extension) to 135° (maximum flexion), limited both physically and by software, with a configurable angular velocity ranging from 0 to 1.5 rad/s. 

Finally, the sensor system integrated into the exoskeleton prototype allows determining the position angle of the servo actuator through an absolute encoder connected to the main drive shaft, the position of the whole structure through MEMS-based sensors, and other parameters related to the operation of the device that can be recorded in real-time for subsequent analysis of the information [47].

### 3.2. Experimental Scenario and Operational Environment

To develop this research, the authors used the available resources in the Human Motion Interactive Laboratory (HMiLab), belonging to the Multisensory Systems and Robotics (SiMuR) research group of the University of Oviedo. This laboratory is a research facility instrumented with cameras, sensors, and a stage that allows the capture of human movements accurately.

The experimental scenario and test environment are composed of 5 elements. It includes (i) an optical motion capture system (also called OTS, Optical Tracking System) based on OptiTrack technology [49], which allows the acquisition of motion data of the robotic exoskeleton prototype structure, its physical behavior, and deformation; (ii) the set of optical tracking markers arranged on the structure to be evaluated; (iii) the robotic exoskeleton prototype described above; and (iv) a wooden life-size upper limb (called dummy arm), which is attached to a fixed support and allows the support the exoskeleton prototype in a suitable way for its evaluation. Finally, (v) a set of test loads is included. 

**Figure 2:**
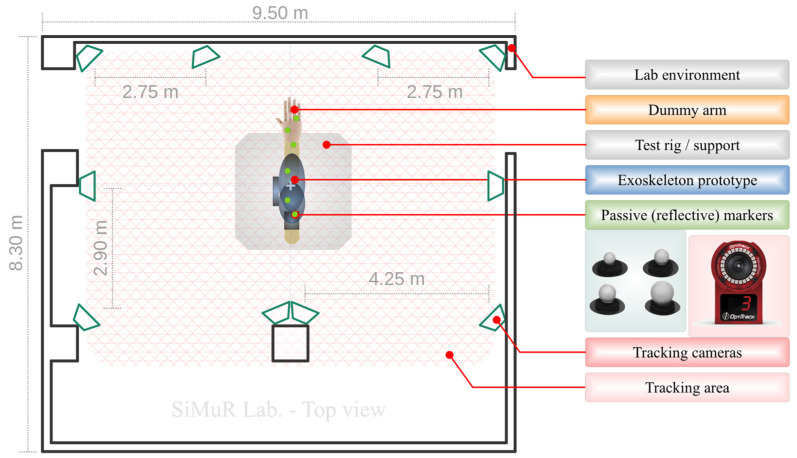
Arrangement of the optical motion capture environment using the OptiTrack Flex 3 cameras, and the other elements of the experimental scenario.

It should be noted that the loads included in the test scenario will act on the arm structure and, ultimately, on the exoskeleton prototype. These loads make it possible to simulate the weight of the human arm and the spasticity that can occur in the rehabilitation process of upper limb motor functions. The dummy arm has a weight intended to simulate a real human arm, and additional external weight is prepared to simulate a spastic condition. This information will be expanded later in the methodology and results concerning the design of the system loads. Figure 2 shown below reflects the arrangement of the experimental scenario.

### 3.3. Optical Tracking System (OTS)

Optical tracking systems (OTSs), also called optical motion capture (OMC) systems, consist of one or more digital sensors, including infrared sensors and highly reliable cameras for tracking the trajectory of moving objects [25,50]. Among their most frequent applications are human body motion analysis for sports applications [51], biomechanical analysis [38], evaluation in mobile robotics [52], and motion analysis for rehabilitation [39]. 

Within the spectrum of technologies covered by OTS, some systems rely on markers, while others do not require them [53]. As mentioned earlier, the equipment used is OptiTrack [49], a commercial system that uses optical markers to capture a visual reference of the object and measure its position in space in three dimensions. 

Specifically, the equipment consists of 10 OptiTrack Flex3 cameras, each with a resolution of 640 × 480 pixels and a sampling rate of 100 FPS. The visible tracking area covered by the cameras arranged in the laboratory is 54 m², distributed in a rectangular shape of 9 m × 6 m, as shown by the grid drawn in Figure 2. 

The cameras are arranged uniformly, allowing effective visibility of the optical markers by at least three cameras at any given time. The test rig, on which the robotic exoskeleton and dummy arm are supported, is located at the center of the tracking area, ensuring that the positioning of the reflective markers is placed almost equidistantly to the tracking cameras.

**Figure 3:**
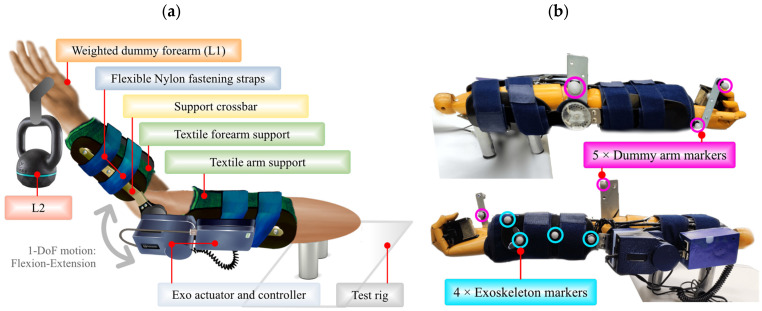
Part (**a**) reflects the layout of the experimental scenario in detail, while part (**b**) shows the arrangement of the optical tracking markers on the elements to be evaluated.

In Figure 3 presented below, specifically in part (a), there is a detailed side view of the system arranged in the experimental scenario, as well as the placement of the optical markers on the structures to be evaluated in this development. The positioning of the optical tracking markers used by the OptiTrack system is performed with two independent sets, as shown in Figure 3b: (i) the first set, composed of 4 optical markers, is placed on the semi-rigid structure of the robotic exoskeleton; while (ii) the second set, composed of 5 additional optical markers, is distributed on the dummy arm. This distribution allows the effective measurement of the movements presented in both the prototype and the dummy upper limb independently. The parameters to be measured in each structure are detailed in Section 4.

**Figure 4:**
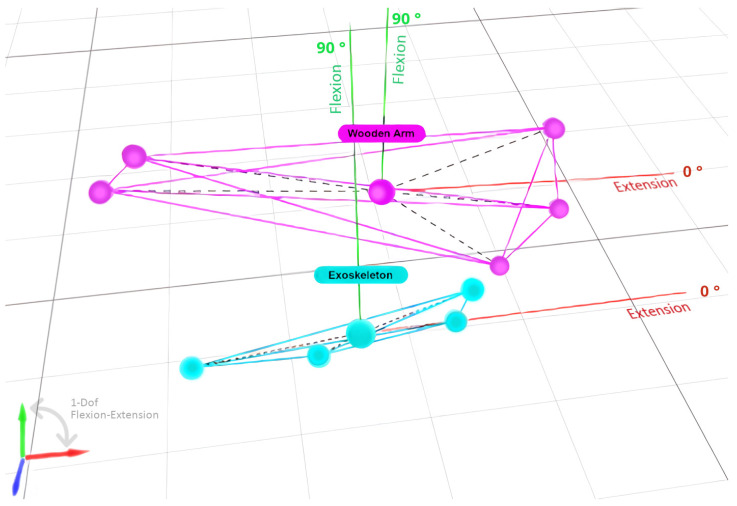
Digitization of the two sets of optical markers using the OTS system, creating two independent Rigid Bodies, corresponding, respectively, to the dummy arm and the robotic exoskeleton prototype.

Using the dual sets of markers and the appropriate configuration in the OTS system management software, two independent rigid bodies are created, whose movement information in space is constantly recorded. Rigid Bodies allow tracking of the motion of a specific structure using different measurement points acting together, as arranged in the experimental scenario. The creation of rigid bodies involves multiple markers on a single structure, and their name is not related to the type of material to be evaluated. Figure 4 below shows the corresponding digitization of the system by tracing the two rigid bodies described above, thus completing the layout of the OTS system in the laboratory.

The relevance of using this OTS system lies in the reliability and accuracy of the measurements that can be obtained. It is emphasized that, with the help of an OTS and the corresponding analysis of the collected data, it is possible to record the deformations of the soft material covering the upper limb, thus obtaining valuable information about the fit, the effective motion transmission, and the assistance in the movement execution for the user rehabilitation.

## 4. Methodology and Experimental Design

The methodology and experimental design of this research focus on the formulation of a test protocol that allows the mechanical evaluation of the robotic exoskeleton, with particular attention to the simulation of upper limb movement in rehabilitation processes. This method was directly derived from the protocol formulated in [54].

The acquisition of motion data through the OTS allows identifying and comparing relevant aspects of rehabilitation with active robots, such as (i) the effective transfer of motion (precision and accuracy) from the prototype to the limb of the human body; (ii) the stability of the prototype structure and the support given to the limb; (iii) the response of the prototype to a possible spastic reaction of the patient, which translates into the active rejection of external disturbances such as the increase in the initial fixed weight; and finally, (iv) the deformation on the flexible and soft structures (textiles) used in the prototype.

The methodological structure of the test protocol is presented below. This protocol guides the analysis of the experimental data collected during the evaluation of the robotic exoskeleton. This process takes several steps, leading to one or more expected results.

### 4.1. Stage 1: Load Design

The load design allows the proper allocation of weight to the dummy arm, allowing adequate stress on the robotic exoskeleton prototype. Furthermore, an additional load is considered to simulate the spasticity (or increased muscle tone) present in some patients in neuromotor rehabilitation processes. Table 1 presented below describes the experimental design and expected outcome corresponding to Stage 1.

### 4.2. Stage 2: Determination of the Effective Transfer of Motion

The purpose of this stage is to qualitatively determine the effective motion transfer from the robotic exoskeleton prototype to the upper limb. Table 2 presented below describes the experimental design and expected outcome corresponding to Stage 2.

### 4.3. Stage 3: Evaluation of Stability and Support in the Upper Limb

This stage aims to quantitatively determine the stability and support given to the upper limb, evaluated as the motion accuracy performed by the robotic exoskeleton. Table 3 presented below describes the experimental design and expected outcome corresponding to Stage 3.

### 4.4. Stage 4: Determination of Response to External Disturbances or Spasticity

This stage aims to determine the response of the exoskeleton prototype (disturbance rejection) when a sporadic and momentary increase in simulated muscle tone is applied, as well as the response of the exoskeleton prototype (reliability in motion) when a continuous increase in muscle tone occurs. Table 4 presented below describes the experimental design and expected outcome corresponding to Stage 4.

## 5. Results

This section contains the results obtained by applying the test protocol defined in the methodological design. The discussion of the results is included in the same subsections, which allows a weighted and comparative analysis.

### 5.1. Stage 1: Load Design

The purpose is to obtain suitable values for (i) the load that simulates the weight of the human arm, and (ii) the removable load to simulate the spastic condition in the rehabilitation process. A total of two independent loads will be designed. The designs are performed theoretically by calculating the average values of the limb weight, which sets the weight of the L1 load. Additionally, the weight of the L2 load is set by the equivalence when the muscle spasticity condition occurs.

For the L1 load sizing, we consider the mean value theoretically determined in [55,56]. The weight of the limb is expressed as a percentage relative to the total weight per person. The sum of the percentage values for the forearm and hand segments is considered since this is the total weight supported by the robotic exoskeleton prototype. To specify a weight, the average biomass value for healthy adults is used, based on the world standard calculated in [57]. The average human body weight (using North America as a reference) is 80 kg. Table 5 below shows these values.

As indicated, the L1 load value is set at 1.8 kg, which is permanently assigned to the dummy arm. This value is an average value for both males and females, also using the average biomass reference value for a healthy adult.

For the L2 load sizing (additional weight exerted in the state of spasticity), it is necessary to determine the relationship between the increase in muscle tone and the weight gain in the upper limb. Following the studies presented in [58,59], it is found that the increase in the average weight of the limbs can be calculated using the torque–weight ratio. By increasing muscle tone, the average torque that can be exerted by the limb is changed; hence, it can be quantified as the perceived average weight on the exoskeleton prototype.

On average, an increase in upper limb torque can occur between 70% and 90% of the nominal value recorded in patients with full muscle control (without spasticity) [58,59]. Considering the above, the L2 load value is set at 2.0 kg. The L2 value is higher than the usual average spasticity increase, which has been set to simulate the worst feasible case. The L2 load is removable and acts on the outside of the dummy arm. Although the torque variation in the spastic condition depends on the angular velocity of the motion [60,61], no variable load design is considered in this research; thus, the value of L2 is fixed for any angular velocity.

### 5.2. Stage 2: Determination of the Effective Transfer of Motion

The objective is to determine the effective motion transfer of the robotic exoskeleton to the limb that is being assisted. Measurements are made to contrast the position of the exoskeleton prototype and the position of the dummy arm through the support structure, quantifying in the process the accuracy of the motion performed. It is emphasized that the dummy arm, including its respective load, is supported by the exoskeleton’s textile (soft) structure, making the motion transfer essential for a proper rehabilitation function.

**Figure 5:**
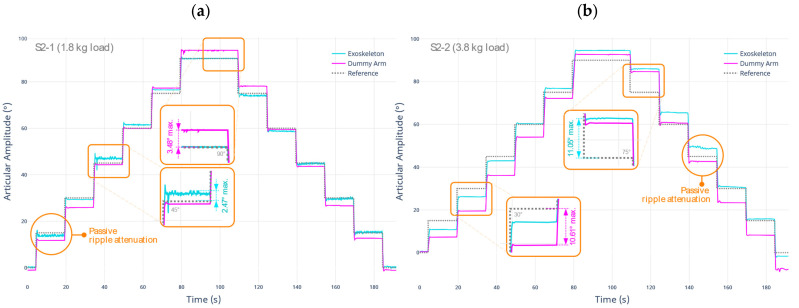
Test result for the determination of effective motion transfer under (**a**) L1 single load equivalent to 1.8 kg; and (**b**) L1 + L2 load equivalent to 3.8 kg.

By applying the test protocol described in S2-1, the stepwise positioning of the prototype at the established references is performed with the L1 load. Then, the same trajectory is performed using the L1 + L2 load to complete the S2-2 test. The results are shown below in Figure 5. It is highlighted that the 0° joint amplitude position corresponds to a full extension of the upper limb, and the values higher than this show the flexion function of the limb.

Concerning the first test protocol (S2-1) using a total load of 1.8 kg, it can be evidenced that the robotic exoskeleton prototype accurately follows the targets in the test. Under normal load conditions, the prototype can reliably reproduce the setpoints produced by the control system. However, a maximum deviation of 2.47° occurs at the most critical motion angle (45°).

Furthermore, the motion produced in the dummy arm can be evidenced as being effective. This is concluded by comparing the similarity in the waveform produced between the motion of the exoskeleton and the motion of the dummy arm. However, a maximum deviation of 3.48° occurs at the critical point (90°). This difference is positive, suggesting that the dummy arm has a greater amplitude than expected. The reason is that the support in the flexion–extension axis is given by the flexible materials of the exoskeleton (wrap-around surfaces), which undergo deformations in both its upper and lower surfaces.

Concerning the second test protocol (S2-2), using a total load of 3.8 kg, it is shown that the robotic exoskeleton prototype does not follow the trajectory, especially at 75°. However, this may be due to an incorrect compensation condition caused by the controller. The controller can recover the reference target for other positions on the trajectory. Likewise, the motion produced in the dummy arm continues close to the target. 

A larger amount of deviation is observed, registering a maximum difference of 10.61°. This amount of error is directly related to the current weight on the limb. This scenario attempts to reproduce the worst-case situation, where muscle tone increase becomes constant, inducing an unnatural motion. However, the motion transfer between the exoskeleton prototype and the dummy arm continues to be within the expected range.

Table 6 shows a summary of the metrics established during the tests developed in this stage. The values were established by comparing the measured value of the rigid body with the OTS system versus the target value.

Considering the magnitude of the RMSE in each test, it is concluded that the motion transfer from the robotic exoskeleton to the dummy arm is effective. In addition, a passive damping effect of the motion ripples was observed in both tests. This means that the use of soft or flexible materials allows higher limb stability by blocking small or vibrating motions originating from the actuator. This condition does not affect the effective transmission of the main movements, as mentioned above.

### 5.3. Stage 3: Evaluation of Stability and Support in the Upper Limb

The goal during this phase is to evaluate the stability and support provided to the upper limb while performing continuous trajectory tracking over time. Measurements will be taken to compare the position of the exoskeleton prototype and the limb and to quantify the accuracy of the motion performed. 

**Figure 6:**
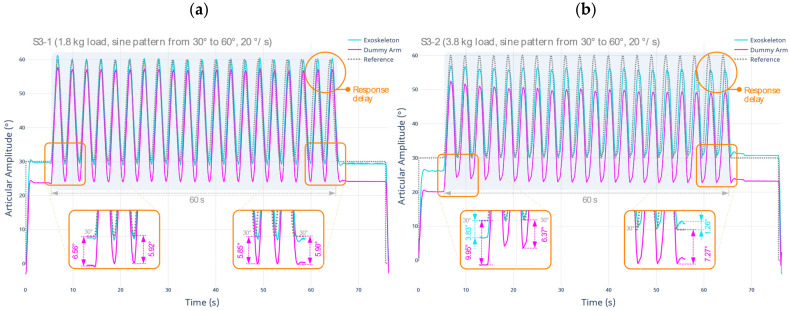
Test result for the evaluation of stability and support in the upper limb, range from 30° to 60°, angular velocity of 20°/s, under (**a**) L1 single load equivalent to 1.8 kg; and (**b**) L1 + L2 load equivalent to 3.8 kg.

By applying the test protocol described in S3-1, the prototype is positioned following a sinusoidal trajectory with the L1 load. Then, the S3-2 protocol is performed, where the same trajectory is repeated now using the L1 + L2 load. The results are shown below in Figure 6.

In the first test protocol (S3-1), using a total load of 1.8 kg, it is evident that the support to the dummy arm is constant. A maximum displacement value of 6.56° occurs, especially at the time of initial positioning. Midway through the test run, the displacement value decreased to a 5.90° average. These measurements are performed on the flexion–extension axis. During the execution of the test, there is no increase in the displacement value produced in the dummy arm; therefore, it is considered that the support to the limb is consistent over time. Regarding the second test protocol (S3-2), using a total load of 3.8 kg, there is evidence that the provided support by the wrap-around surfaces is slightly lower. First, there is an increase in the maximum displacement value, standing at 9.95°. It is indicative of a higher deformation of the flexible material. Second, the support provided to the dummy arm is not constant, starting at an average value of 6.37° and ending near 7.27°. 

This implies that an increased load on the prototype will cause higher structure deformations, which are also dependent on the activity time of the exoskeleton prototype. However, it is concluded that the support performance is satisfactory, considering that this test simulates the worst spasticity scenario, where the exoskeleton prototype is able to support the weight of the arm and the increased muscle tone without a substantial amount of deformation. The performance of the intended motion trajectories is fully executed in any scenario, satisfactorily fulfilling the motion assist function. A summary of the metrics established for the developed tests is presented in Table 7 below.

Two additional important factors are highlighted. (i) In either of the applied protocols, both S3-1 and S3-2, the robotic exoskeleton achieves the expected reference values, with a maximum deviation of 3.83°. As anticipated, the positioning accuracy of the exoskeleton is compromised to a greater extent using the additional spastic load (L1 + L2). 

However, the absolute deviation versus the reference value is minimal under normal operating conditions of the device (only using L1). (ii) The controller may slightly delay the trajectory tracking temporarily, resulting in a lag in the ideal response time. This delay does not affect the performance of the device, although it increases the average error of the exoskeleton positioning, thus mainly impacting the RMSE metric.

**Figure 7:**
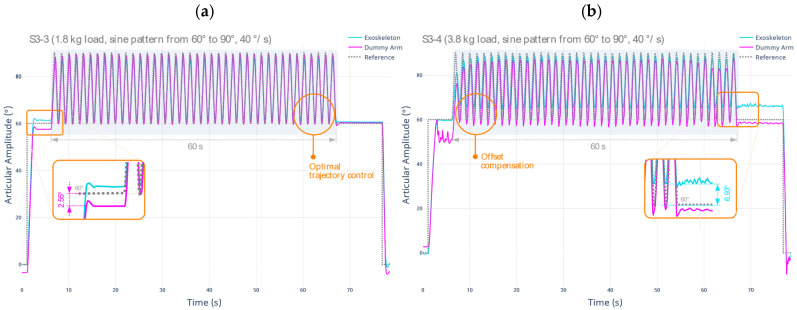
Test result for the evaluation of stability and support in the upper limb, range from 60° to 90°, angular velocity of 40°/s, under (**a**) L1 single load equivalent to 1.8 kg; and (**b**) L1 + L2 load equivalent to 3.8 kg.

By applying the test protocol described in S3-3 and S3-4, additional tests are performed regarding the stability of the dummy arm provided by the exoskeleton prototype, now considering a different joint amplitude range and a higher angular velocity. The results are shown below in Figure 7.

This behavior may be due to the weight of the dummy arm no longer being influenced vertically by gravity, resulting in less deformation of the flexible support material. A maximum displacement compared to the target of 2.55° occurs, especially at initial positioning. Midway through the test run, the displacement value in the position decreased to an average of 1.83°.

Regarding the fourth test protocol (S3-4), using a total load of 3.8 kg, there is evidence that the support given is lower compared to the previous result. However, the overall performance is better. Unlike the previous test, the controller of the robotic exoskeleton prototype compensates to a greater extent for the displacements limb to continue an optimal trajectory tracking. Due to a larger number of oscillations induced by the additional weight, the movement of the exoskeleton differs in amplitude to the setpoint, causing a deviation of at least 6.93°. However, the execution of the planned trajectories is fully executed in any scenario, satisfactorily fulfilling the function of assisting the movement of the limb. Table 8 below summarizes the established metrics for the described tests.

Considering the metrics shown in this stage, it is concluded that the upper limb stability and support are satisfactory. This is highlighted when flexible materials are used for the main support of the limb. In addition, a better response of the system is shown in the range from 60° to 90°, where the average errors are lower when compared to the 0° to 60° motion range. Similarly, it is concluded that the angular velocity does not appreciably affect the support properties, allowing the device to be used safely under all circumstances.

### 5.4. Stage 4: Determination of Response to External Disturbances or Spasticity

The goal of this stage is to determine the prototype’s response to external perturbations or spasticity. A trajectory tracking routine will be performed, using discrete as well as continuous values over time. It is reiterated that spastic reactions under this evaluation approach can be evidenced as an increase in the natural weight of the arm in the same direction of movement.

**Figure 8:**
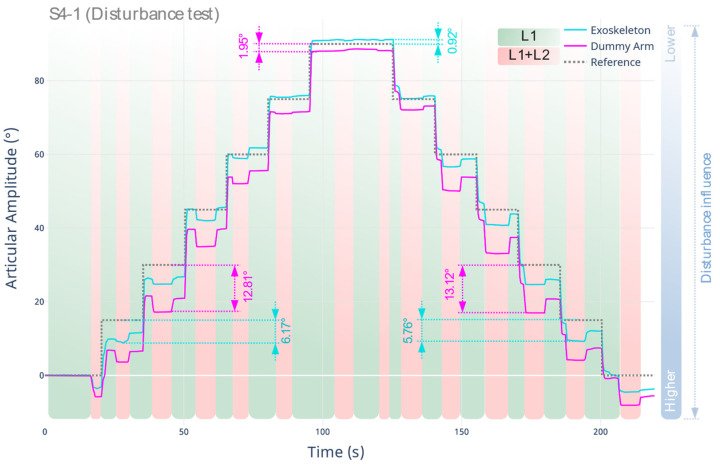
Response of the robotic exoskeleton prototype to external perturbation when the target is reached, according to the S4-1 protocol.

By applying the test protocol described in S4-1, the response of the system to an external disturbance is measured when a specific target is reached. In this case, L2 is added to the dummy arm at the setpoint reached, measuring the generated limb displacement and the deformation in the robotic exoskeleton. The results are shown below in Figure 8.

The disturbance has a greater degree of influence at smaller joint amplitude angles (full extension of the upper extremity). This is due to the arrangement of the loads, where the dummy arm, affected by gravity, transfers the load entirely to the soft support of the exoskeleton vertically. As the limb flexes, the component is not purely vertical. Therefore, the addition of L2 at 90° has a lesser impact compared to the load addition between 0° and 45°. Additionally, when L2 is added at each setpoint, a maximum displacement value of 13.12° is recorded at the dummy arm. This is caused by the initial displacement of the actuator, which does not comply with the expected target, as well as the additional deformation caused by the materials used in the limb support. The maximum deflection of the exoskeleton (actuator) from the reference when L2 is added is 6.17°.

**Figure 9:**
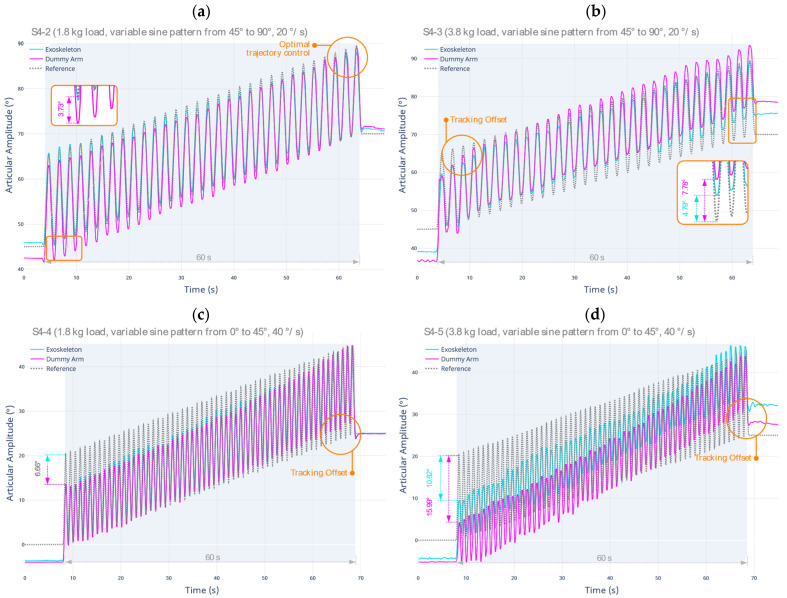
Response of the system to external disturbances produced in the upper limb, under sine sweep trajectory using (**a**) L1 load from 45° to 90°, 20°/s; (**b**) L1 + L2 load; (**c**) L1 load from 0° to 45°, 40°/s; and (**d**) L1 + L2 load.

For a comprehensive assessment, the tests described in S4-2 through S4-5 are performed. In those cases, the external disturbances occur continuously over time and under worst-case scenario conditions. These tests are designed to perform a full sweep of the joint amplitude range, now with an ascending variable sinusoidal pattern and at two different angular velocities. The results are shown below in Figure 9.

Regarding the second test protocol (S4-2), using a total load of 1,8 kg, deformations occur in the support early in the test. The maximum deviation of the dummy arm position from the reference is 3.73° maximum. The overall response of the system to disturbances is satisfactory since optimal trajectory tracking is maintained until the test completion. For the third test protocol (S4-3), using a total load of 3.8 kg following the same sweep sequence as before, a higher deformation amount is obtained in the flexible support, resulting in a lower response of the system to disturbances with a high load. In particular, the maximum deviation of the position of the dummy arm from the reference is 7.78°, while the maximum deviation of the robotic exoskeleton is 4.79°. However, the overall response of the system to perturbations continues to be satisfactory, maintaining the shape of the programmed movement trajectory without any time or response lag.

Concerning the fourth test protocol (S4-4), a maximum deviation of 6.66° is produced, both for the dummy arm and the robotic exoskeleton prototype. Despite not reaching the desired reference, trajectory tracking is maintained until the conclusion of the test, albeit showing variability in the achieved joint amplitude. 

Both protocols place maximum demands on the system since the loads induce disturbances in both the top and bottom of the wrap-around surfaces. The oscillations in these soft support structures try to be actively compensated by the controller, where, finally, no further deformation of the structures is evidenced. 

Regarding the fifth test protocol (S4-5), using a total load of 3.8 kg and following the same sweep sequence of the previous test, considerable deformations occur in the support structures, combined with deviations in the robotic exoskeleton position. Concretely, there is a maximum deviation of the dummy arm position of 15.99°. The maximum position deviation of the robotic exoskeleton is 10.82°. Although these maximum values are high in both cases, there is no increased deformation of the supporting structures, since the position of the actuator has a pronounced phase shift when tracking the peak values of the trajectory. Table 9 and Table 10 below summarize the metrics established for the tests developed and described previously.

It is concluded from the results obtained at this stage that the prototype has medium tolerance to external perturbations, being able to reject or counteract some of them. Finally, it is highlighted that two performance characteristics are retained in any scenario: (i) the trajectory shape tracking is constant; and (ii) the flexible/textile support structures used in this development, although they suffer different magnitude deformations under spastic conditions, fully comply with the complete support of the upper limb. This allows the motion transfer from the robotic exoskeleton in a safe way.

## 6. Conclusions

This paper describes the protocol and application of specific tests to an upper limb exoskeleton prototype oriented to rehabilitation processes, seeking the evaluation of some of its mechanical properties using an optical motion capture (OTS) system within a controlled laboratory environment. By applying the established protocol, it is possible to characterize relevant aspects such as the effective motion transfer, the stability of the prototype structure, the response to external disturbances, and the deformation of the support surfaces, since textile, soft or flexible materials are being used.

The results demonstrate the effective motion transfer from the robotic exoskeleton to the limb. Using flexible materials to support critical limb weight does not override the effectiveness of the robotic actuator in inducing or counteracting movements to the limb, which is desired in all types of applications. 

An additional effect, not planned but beneficial to the rehabilitation process, is an additional passive effect that dampens the ripple in motion caused by the mechanical coupling used and increases the limb stability as minor vibrations are not transmitted.

The stability analysis and the support provided in the upper limb concerning motion tracking are satisfactory for this case study. A lower number of oscillations or internal displacements of the limb is evidenced in the range from 45° to 90°. On average, the initial range from 0° to 45° registers a higher limb displacement or positioning error values. Likewise, it is concluded that the angular speed does not noticeably influence the support characteristics of the upper limb, which allows the device to be used safely in all circumstances. 

It is concluded that the prototype has medium tolerance to external disturbances, rejecting or counteracting some of the disturbances. However, although the deformation amount of the soft support structure is higher than in other types of tests, the essential performance characteristics of the prototype robotic exoskeleton are preserved in any scenario. The tracking of the path shape is secured, and the support structures are fully functional in supporting the upper limb.

It should be noted that while this study provides an evaluation methodology based on test loads, the same protocol may be used in the future when the prototype robotic exoskeleton is used on the human body. The deformations of the materials used are expected to be consistent with those demonstrated in these tests under similar conditions inside or outside the laboratory. However, there may be some variations due to the dynamic behavior of the natural upper limbs, such as additional extensions or contractions beyond those described here. 

It is emphasized that this testing protocol, along with a rigorous analysis of the collected data, can guide future actions to develop better robotic assistance systems. Such improvements in the design of future devices will not only deal with ergonomics, weight reduction, and complexity but also with performing efficient movements for the intended task. 

Finally, one of the most important contributions of this research is the identification of a versatile protocol that can be used as a basic guide for other types of mechanical evaluations in the laboratory. The materials and methods used can be modified and adapted according to the needs of each case, which allows exploring new alternatives to mechanical evaluation. 

It is highlighted that, independently of the actuating technique used on the robotic exoskeleton prototype, the application of this protocol or its variations will provide useful information about the mechanical behavior that occurs specifically in the soft or textile parts that support the limbs of the human body on which they operate, allowing a deeper evaluation of the physical design of this type of motion assistance systems.

## Figures and Tables

**Table 1:** Experimental design and expected outcome corresponding to Stage 1.

Steps	Detailed Description
S1-1	Determination of the average natural weight of the upper limb of the human body, specifically the forearm section, for adult male and female subjects.
S1-2	Determination of the variation in muscle tone, perceived as a variation in arm weight (load increase), for the same population group described in S1.1.
**Collected Data**	**Analysis/Expected Outcome**
S1-1, S1-2: Anthropometric measurements (weight) [Theoretical data].	S1-1: Artificial load (labeled as L1) with an average net weight according to the human body anatomy. S1-2: Artificial load (labeled as L2) with an average net weight according to the increase in muscle tone (spasticity).

**Table 2:** Experimental design and expected outcome corresponding to Stage 2.

Steps	Detailed Description
S2-1	Motion type: References established at fixed points with an articular amplitude of 0°, 15°, 30°, 45°, 60°, 75°, and 90°. Cycles: Once in each position, in an ascending and descending direction. Load type: L1.
S2-2	Motion type and cycles: As described in S2-1. Load type: L1 + L2.
**Collected Data**	**Analysis/Expected Outcome**
S2-1, S2-2: Joint amplitude in degrees. S2-2: Deformation, displacement or misalignment using OTS.	The results obtained both in S2-1 (baseline) and S2-2 are contrasted, establishing the amount of error using RMSE, providing a conclusion about the effective motion transfer.

**Table 3:** Experimental design and expected outcome corresponding to Stage 3.

Steps	Detailed Description
S3-1	Motion type: Sinusoidal pattern with joint amplitude between 30° and 60°, angular velocity of 20°/s. Length: 60 s. Load type: L1.
S3-2	Motion type and length: As described in S3-1. Load type: L1 + L2.
S3-3	Motion type: Sinusoidal pattern with joint amplitude between 60° and 90°, angular velocity of 40°/s. Length: 60 s. Load type: L1.
S3-4	Motion type and length: As described in S3-3. Load type: L1 + L2.
**Collected Data**	**Analysis/Expected Outcome**
From S3.1 to S3.4: Joint amplitude in degrees. S3-2, S3-4: Deformation, displacement or misalignment using OTS.	The results obtained both in S3-1 and S3-3 (baseline) and in the motions with load performed in S3-2 and S3-4 are contrasted, respectively, establishing the amount of error using RMSE, concluding about the stability and support in the upper limb.

**Table 4:** Experimental design and expected outcome corresponding to Stage 4.

Steps	Detailed Description
S4-1	Motion type: References established at fixed points with an articular amplitude of 0°, 15°, 30°, 45°, 60°, 75°, and 90°. Cycles: Once in each position, in an ascending and descending direction. Load type: L1, then L1 + L2 when the reference is reached.
S4-2	Motion type: Sinusoidal pattern with ascending joint amplitude between 45° and 90° with oscillations of 20°, angular velocity of 20°/s. Cycles: Once. Load type: L1.
S4-3	Motion type and cycles: As described in S4-3. Load type: L1 + L2.
S4-4	Motion type: Sinusoidal pattern with ascending joint amplitude between 0° and 45° with oscillations of 20°, angular velocity of 40°/s. Cycles: Once. Load type: L1.
S4-5	Motion type and cycles: As described in S4-4. Load type: L1 + L2.
**Collected Data**	**Analysis/Expected Outcome**
From S4-1 to S4-5: Joint amplitude in degrees. S4-1, S4-3, S4-5: Deformation, displacement or misalignment using OTS.	In S4-1, the results before and after adding the L2 load at each setpoint are contrasted. The rejection of disturbances is evaluated using the RMSE metric. The results obtained in S4-2 and S4-4 (baseline) and the motions with additional load in S4-3 and S4-5 are contrasted, respectively, concluding about the response of the prototype to external disturbances or spasticity.

**Table 5:** Theoretical estimation of the weight of the forearm and hand segments for the human body.

Segment	Male [55]	Male [56]	Female [55]	Female [56]	Avg.	Avg. Weight
Forearm	1.87%	1.62%	1.57%	1.38%	1.61%	1.3 kg
Hand	0.65%	0.61%	0.50%	0.56%	0.58%	0.5 kg
Forearm and Hand	2.52%	2.23%	2.07%	1.94%	2.19%	1.8 kg

**Table 6:** Summary of positioning errors under Stage 2 tests.

Rigid Body	S2-1		S2-2	
	Max. Error	RMSE	Max. Error	RMSE
Exoskeleton	2.47°	1.52°	11.05°	4.78°
Dummy Arm	3.48°	2.90°	10.61°	6.71°

**Table 7:** Summary of the stability and support in the upper limb test metrics under S3-1 and S3-2.

Rigid Body	S3-1			S3-2		
	Max. Pos. Error	Positioning RMSE	Max. Displacement	Max. Pos. Error	Positioning RMSE	Max. Displacement
Exoskeleton	1.13°	2.64°	N/A	3.83°	2.89°	N/A
Dummy Arm	6.56°	7.48°	17.67 mm	9.95°	10.39°	28.81 mm

**Table 8:** Summary of the stability and support in the upper limb test metrics under S3-3 and S3-4.

Rigid Body	S3-3			S3-4		
	Max. Pos. Error	Positioning RMSE	Max. Displacement	Max. Pos. Error	Positioning RMSE	Max. Displacement
Exoskeleton	1.72°	0.79°	N/A	6.93°	7.96°	N/A
Dummy Arm	2.55°	1.83°	6.86 mm	10.31°	8.19°	20.98 mm

**Table 9:** Summary of disturbance rejection test metrics under S4-2 and S4-3.

Rigid Body	S4-2			S4-3		
	Max. Pos. Error	Positioning RMSE	Max. Displacement	Max. Pos. Error	Positioning RMSE	Max. Displacement
Exoskeleton	1.02°	0.81°	N/A	4.79°	2.78°	N/A
Dummy Arm	3.73°	2.35°	7.7 mm	7.78°	4.45°	9.23 mm

**Table 10:** Summary of disturbance rejection test metrics under S4-4 and S4-5.

Rigid Body	S4-4			S4-5		
	Max. Pos. Error	Positioning RMSE	Max. Displacement	Max. Pos. Error	Positioning RMSE	Max. Displacement
Exoskeleton	6.66°	3.67°	N/A	10.82°	5.57°	N/A
Dummy Arm	6.66°	3.90°	2.99 mm	15.99°	7.18°	19.95 mm

## Data Availability

Not applicable.
